# Dynamics of bird assemblages in response to temporally and spatially variable resources in arid Australia

**DOI:** 10.1002/ece3.7293

**Published:** 2021-03-17

**Authors:** Bruce A. Pascoe, Chris R. Pavey, Stephen R. Morton, Christine A. Schlesinger

**Affiliations:** ^1^ Research Institute for the Environment and Livelihoods Charles Darwin University Alice Springs NT Australia; ^2^ Alice Springs Desert Park, Department of Environment, Parks and Water Security Alice Springs NT Australia; ^3^ CSIRO Land and Water Darwin NT Australia

**Keywords:** arid‐zone, birds, boom and bust, desert, rainfall, resource pulse

## Abstract

Bird assemblages in arid Australia are often characterized as being highly variable through time in response to boom and bust dynamics, although the importance of habitat in structuring assemblages at a local‐scale is also recognized. We use a novel approach to investigate the importance of rainfall variability in structuring bird assemblages in a resource‐limited environment. Monthly bird surveys were conducted at ten plots for 8 years at a botanical and zoological park in central Australia, including five irrigated plots within a fenced area and five natural plots outside. Irrigation—used to promote growth, flowering, and fruiting of plants—created an artificial resource‐enhanced environment against which the response of birds to natural fluctuations in season and rainfall were compared. Species richness was generally maintained at a higher level in resource‐enhanced plots during dry times but was higher in natural plots when rainfall was high, mainly due to increases in granivores and insectivores. Honeyeaters were consistently more abundant at irrigated plots. Rainfall was important in structuring bird assemblages at all plots; however, assemblages were more stable in irrigated plots and did not respond as dramatically to a period of very high rainfall. The comparative smoothing of fluctuations in the composition and abundance of birds in irrigated areas highlights the importance of primary productivity, normally tied to rainfall, in driving temporal change in arid‐zone bird communities. There was also evidence that different plots in differing habitats supported distinct bird assemblages and that this spatial distinctiveness persisted irrespective of rainfall and determined, to some extent, the response to rainfall. Our study is one of few long‐term studies of arid bird assemblages and highlights the importance of both long‐term cycles of productivity driven by rain and season as well as site differences in the dynamics of arid‐zone bird communities. These insights are particularly valuable as climate change further exacerbates rainfall variability worldwide and initiatives to conserve avifauna in increasingly extreme environments may be required.

## Graphical abstract

Arid‐zone bird assemblages in natural areas were contrasted with areas where resources were enhanced by artificial irrigation over 8 years under average to dry conditions and including a time of very high rainfall. Rainfall was important in structuring bird assemblages and a comparative smoothing of fluctuations in the composition and abundance of birds in irrigated areas highlights the importance of primary productivity in driving temporal change in arid‐zone bird communities.

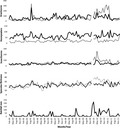

## 1 INTRODUCTION

Rainfall is one of the dominant drivers of arid ecosystems (Morton et al., 2011). Resource pulses in arid environments are usually triggered by the supply of water, which varies through space and time to a degree much greater than in most other environments (Chesson et al., 2004; Van Etten, 2009). This variation in the availability of water determines the amount of energy available in a system and can, in turn, influence the biota that can be supported at a particular place and time. Thus, in a local area, vertebrate faunal assemblages can be constrained by fluctuations in rainfall which determine productive output from plants including flowering, new growth and seeding, and parallel and consequential changes in abundance of invertebrate prey (Nano & Pavey, 2013; Schwinning & Sala, 2004). For birds, these temporal effects may be manifested in the presence or absence of nomadic species or seasonal migrants that have moved into an area to take advantage of available resources and in an increase in abundance of both resident and mobile species through successful breeding (Davies, 1984; Dean et al., 2009; Jordan et al., 2017).

Floristic composition and habitat structure similarly are recognized as playing a pivotal role in determining the makeup of avifaunal assemblages in arid environments and elsewhere (Antos & Bennett, 2005; Cody, 2001; Ferger et al., 2014; Fleishman et al., 2003; Lindenmayer et al., 2010), and even fine‐scale variation in habitat structure and composition is likely to influence habitat use (Maguire, 2006). This is demonstrated, for example, through the increase or decrease in abundance of species in response to habitat disturbance (Abbott et al., 2003), and changes to local biodiversity in response to changes in land‐cover (Acevedo & Restrepo, 2008). In short, primary productivity and spatial heterogeneity of environmental resources are both important determinants of species presence at a local‐scale (Bailey et al., 2004) and the relationship between the composition and abundance of avian assemblages with habitat must also be considered when examining the influence of temporal pulses of production.

Quantifying the effects of variation in resources on the composition of faunal assemblages and species abundances in arid regions such as central Australia is highly challenging. The unpredictability of rainfall and resulting variation in the occurrence of resource pulses make it logistically difficult to design and implement before–after control‐impact experiments (Wardle et al., 2013). Further, the remoteness and limited human resources and infrastructure in much of arid Australia make it difficult to monitor populations over the lengthy timeframes that are required to compare times of least production with the infrequent resource pulses caused by rainfall. Many bird surveys in Australia are run for <6 months (Burbidge & Fuller, 2007; Cody, 1994; Paltridge & Southgate, 2001; Recher, 1984) but much longer time‐scales are necessary to capture a range of representative climatic conditions in arid regions, where rainfall is infrequent (Jordan et al., 2017; Lindenmayer et al., 2012).

Here, we overcome these logistical challenges by taking advantage of an irrigated desert environment within a botanical and zoological park in central Australia to examine the impact of resource pulses on bird assemblages in the broader landscape. We contrast the temporal dynamics of bird assemblages from the irrigated environment, where water availability is relatively consistent over time, with those of the adjacent natural desert environment that is fully exposed to the impacts of rainfall unpredictability, using 8 years of monthly survey data collected by citizen scientists.

We predicted that the vegetative response to irrigation, which supplements natural rainfall, would lead to ongoing increased availability of both plant and animal food resources for birds, and this would produce a buffering effect on natural boom‐bust cycles with fluctuations in bird assemblages being reduced in comparison to nonirrigated sites. There is some evidence that natural areas with relatively high residual groundwater can support more stable or resistant avifaunal communities during drought than surrounding areas (Selwood et al., 2015) and we expected similar but stronger effects at artificially irrigated sites. Aside from testing this key hypothesis, by also considering the differing vegetation structure and floristics of survey sites within the irrigated and natural environments and examining how consistent bird assemblages are through time, especially at natural plots, we contrast the impacts of climatic cycles on local bird assemblages with spatial patterning. Thereby, we tested the proposition – based on a bioregional‐scale study by Pavey and Nano (2009) — that vegetation type is an important influence on the bird assemblage as well as temporal dynamism.

We use data collected monthly from 2004 to 2011 to test our hypotheses about temporal change in avian assemblages in response to rainfall and the degree to which bird assemblages correspond to habitat. The questions addressed by the study are:

1. Are there differences between the components and rates of temporal change in avian assemblages in irrigated and natural areas? We predicted that birds in natural areas would be limited by the availability of food resources, especially during dry times, and conversely that birds would be more abundant and species richness would be higher in the irrigated area at most times due to higher primary productivity and the buffering effect of irrigation on climatic variability.
2. How do avian assemblages change through time, and in response to what factors? Focusing predominantly on the natural sites we predicted that temporal changes, such as species irruptions or arrival of mobile species, would occur in response to temporally unpredictable resource pulses, and be superimposed on regular variation in avian assemblages caused by seasonal resource availability.
3. Do the different habitats at our study plots support distinct bird assemblages? We predicted that assemblages of birds at different survey plots would be distinct and retain their distinctiveness over time.

## 2 METHODS

### 2.1 Study site

The Alice Springs Desert Park is located six km southwest of the centre of Alice Springs, Northern Territory, Australia. The climate is characterized by high daytime temperatures (mean max temperature >30°C) for 6 months of the year (October–March), low nighttime temperatures (mean minimum temperature <10°C) during a short winter (May–August) and low (Mean annual rainfall = 283 mm) and highly variable and unpredictable rainfall that can fall at any time of the year (http://www.bom.gov.au, summary statistics Alice Springs Airport).

The Desert Park consists of a 50 ha core area enclosed by a fence designed to exclude feral predators, surrounded by a 1,300 ha protected area of unmodified vegetation. Approximately, 30 ha of the enclosed area comprises an irrigated botanic garden representing key habitats of inland Australia and are open to public access on walking paths (Land Systems EBC, 1994).

### 2.2 Resource availability in the irrigated and surrounding areas

The development of the Desert Park site began in 1996 with planting of native vegetation to recreate distinctive central Australian plant communities in different areas. The existing vegetation was changed structurally and floristically in some areas and retained—at least partially—in others depending on the habitat being represented. Irrigation is used throughout the planted areas to assist in establishing new plants and encourage growth, flowering, and fruiting irrespective of natural climatic conditions. As well as natural rainfall the irrigated area receives on average the equivalent of 64 mm of rainfall per month, or 750 mm per year, delivered through dripper systems which run for 8 h every fortnight (Gary Dinham, personal communication). On average, therefore, the irrigated area receives moisture exceeding twice the annual average rainfall—equivalent to what could be considered a boom year in central Australia—but spread evenly throughout the year. Monthly irrigation exceeds mean monthly rainfall for all months and maintains soil moisture at levels that ensure flowering, fruiting, and seeding of plants is maximized throughout the year (Friedel et al., 1993). In contrast, the area surrounding the irrigated area of the park is reliant on natural rainfall only and is unmanaged except for fencing to exclude grazing animals and systematic slashing of fire breaks to reduce continuity fuel loads of *Cenchrus ciliaris*, an invasive high biomass grass that is dominant in the area.

### 2.3 Vegetation communities and survey methods

**FIGURE 1:**
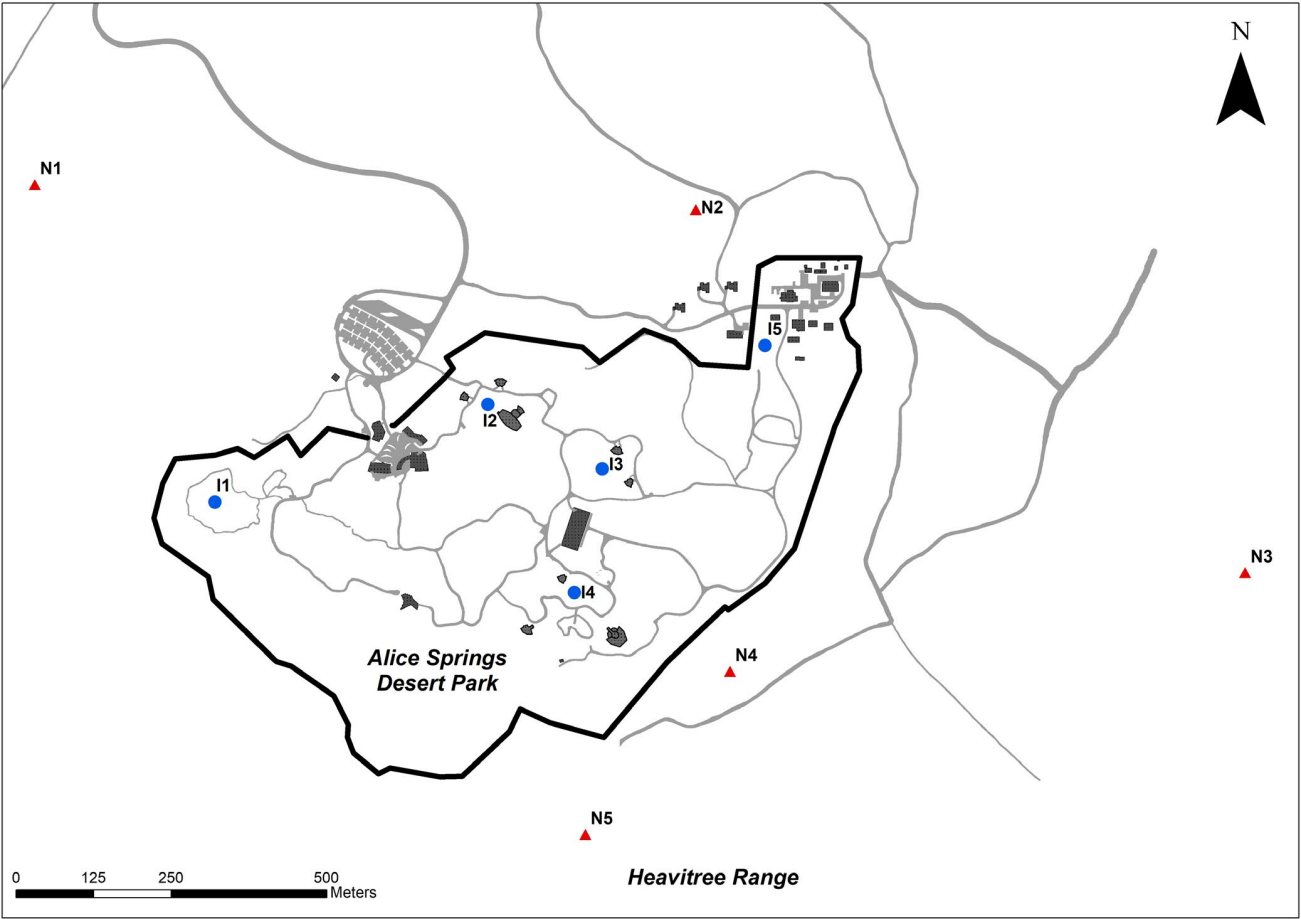
Location of survey plots within the irrigated area (I1–I5, blue circles) and in surrounding natural areas (N1–N5, red triangles) at the Alice Springs Desert Park. The perimeter fence of the park is indicated in black and walking tracks and access roads inside and outside the fenced area are shown in grey. Major infrastructure (buildings) are depicted

Five survey plots in each of the irrigated (I1–I5) and nonirrigated (natural) areas (N1–N5) of the park were monitored. The irrigated plots included four areas of the Park used for public display and one area of amenity planting with no public access. Plots were chosen to represent the variety of vegetation types inside and outside the Park (Table 1; Figure 1). The vegetation communities inside the Park are similar to natural habitats in close proximity outside it (as described by Albrect & Pitts, 2004), except (a) plot I3, an artificial construction of a spinifex community planted on red sand soils transported to the site, does not correspond with any natural habitats nearby and (b) introduced *Cenchrus ciliaris* (buffelgrass), dominated the understory of all the natural rainfall plots but had been removed from all irrigated areas. Plot boundaries inside the Park were based around habitat plantings and access paths and were thus irregular but were approximately two hectares in size. Plots in nonirrigated areas were also approximately two hectares in size and rectangular (100 m × 200 m) in shape.

**TABLE 1:** Vegetation and habitat characteristics at survey plots

Plot	Vegetation description	Prominent species	Average height of overstorey (m)	Cover of shrubs and trees (%)
I1	Planted *Acacia* shrubland on gravelly rises of granite, gneiss, schist, or quartz	*Acacia aneura* *Acacia kempeana* *Eremophila* spp. *Hakea lorea*	6	70
I2	Planted river red gum woodland and coolabah swamp on Rocky/sandy creekline	*Acacia jennerae* *Eucalyptus camaldulensis* *Eucalyptus coolabah* *Melaleuca bracteata*	20	80
I3	Planted mixed communities that occur in red sand areas: mallee, mulga, spinifex, melaleuca. Open grass and shrubland areas on red sand and clay with underlying gneiss or schist	*Acacia aneura* *Acacia cyperophylla* *Acacia ligulata* *Eremophila* spp. *Eucalyptus gamophylla* *Eucalyptus pachyphylla* *Grevillea eriostachya* *Grevillea juncifolia* *Melaleuca glomerata* *Triodia* spp.	8	60
I4	Planted with some naturally occurring larger trees. Mixed open woodland communities; hakea, ironwood, eucalypts Open grass and shrubland areas on granite, gneiss or schist	*Acacia estrophiolata* *Acacia tetragonophylla* *Atalaya hemiglauca* *Corymbia opaca* *Eremophila* spp. *Eucalyptus thozetiana* *Hakea divaricate* *Hakea leucoptera*	15	50
I5	Naturally occurring *Acacia* shrubland and open grassland supplemented with plantings on alluvial flats.	*Acacia estrophiolata* *Acacia kempeana* *Corymba opaca* *Grevillea striata* *Hakea lorea*	10	30
N1	Naturally occurring witchetty bush or mulga on gravelly rises of granite, gneiss, schist, or quartz and ironwood and fork‐leaved corkwood on alluvial flats.	*Acacia estrophiolata* *Atalaya hemiglauca* *Acacia aneura* *Acacia kempeana* *Cenchrus ciliaris*	15	20
N2	Rocky/sandy creekline with tea tree and redgum	*Acacia estrophiolata* *Acacia kempeana* *Cenchrus ciliaris* *Eucalyptus camaldulensis* *Melaleuca bracteata*	20	20
N3	Ironwood and fork‐leaved corkwood on alluvial flats	*Acacia estrophiolata* *Acacia victoriae* *Atalaya hemiglauca* *Cenchrus ciliaris* *Hakea divaricata*	10	15
N4	Witchetty bush and mulga on rocky hills of granite, gneiss or schist	*Acacia aneura* *Acacia kempeana* *Cenchrus ciliaris* *Hakea divaricate* Hakea lorea	6	50
N5	Mulga on rocky slopes of quartzite, sandstone or silcrete	*Acacia aneura* *Cenchrus ciliaris* *Eremophila freelingii* *Senna* spp.	8	70

The proximity of plots, especially in the irrigated area (Figure 1), meant that some movement of birds between plots was inevitable and surveys at different plots could not be considered independent. However, all surveys were conducted simultaneously to reduce the possibility of birds being counted on multiple plots in one survey period and only birds that were actively using the plot, rather than transiting, were recorded. Therefore, our results reflect the birds that were using each plot during the surveys rather than the bird assemblage present in the broader area.

The 10 plots were each surveyed on 94 occasions between February 2004 and December 2011, normally on the first Wednesday of each month. The assistance of volunteers enabled all 10 plots to be sampled simultaneously on each occasion.

Surveys were sometimes conducted by more than one person per plot. On these occasions, participants would stay together to view each species detected. There were also occasions when insufficient participants were available, and then another participant would survey a second plot immediately after finishing the first. Surveys were postponed several times due to weather; but only once, in January 2010, was the survey of all plots canceled due to lack of participants. All volunteers were keen bird observers but their level of experience with surveys varied, thereby introducing a level of observer bias into this study. It is likely, for example, that some of the smaller and more cryptic species, or species that are difficult to identify, may have been missed in some surveys or plots if an observer was less experienced. This was considered in the data analysis by focusing on broad trends and functional groups rather than patterns for individual species.

Surveys began at 07:00 in summer, and 08:30 in winter (when early morning temperatures were often below 5°C). The survey was a 30‐min timed area‐search in which participants walked through the plot (without retracing steps), recording the number of individuals of each species seen within each plot.

### 2.4 Data analysis

Due to issues associated with nonindependence of plots, irrigated plots all being within one area, and observer bias, our analyses focus on broad patterns of temporal change in natural compared with irrigated areas (i.e., species richness, abundance of functional groups, and broad compositional changes) followed by a more detailed examination of temporal patterns in natural areas. Spatial patterns across all plots are interpreted cautiously, again with a primary focus on the natural plots which were spatially independent.

Spatial and temporal variation between bird assemblages was explored and analyzed using Primer‐e V7 Ltd and PERMANOVA+ (Anderson et al., 2008). Data were log (*x* + 1) transformed to reduce the impact of abundant species and the Bray–Curtis similarity coefficient was computed to develop a resemblance matrix. Principal coordinate's analysis (PCO) and nonmetric multidimensional scaling (nMDS) were also used to visualize the data.

The null hypothesis in each case was tested using permutational multivariate analysis of variance (PERMANOVA) with fixed factors of Year, Season, and Irrigation (irrigated/natural) with random factors Plot nested in Irrigation and Month nested in Year. The contributions of individual species to similarities within and differences between groups from the nMDS were examined using similarity percentages (SIMPER).

Parametric (Pearson) correlations were undertaken in Minitab 16 (Minitab Inc.) to examine the relationship between rainfall and species abundance at natural plots. Cumulative rainfall was calculated over six time periods (1, 2, 3, 4, 5, 6 months), and correlated with total bird abundance and abundance of different functional groups (nomadic and resident species; and for three feeding guilds, granivores, insectivores and honeyeaters following classifications in Pavey & Nano, 2009).

## 3 RESULTS

### 3.1 Temporal change in bird assemblages in relation to rainfall in irrigated and natural areas

**FIGURE 2:**
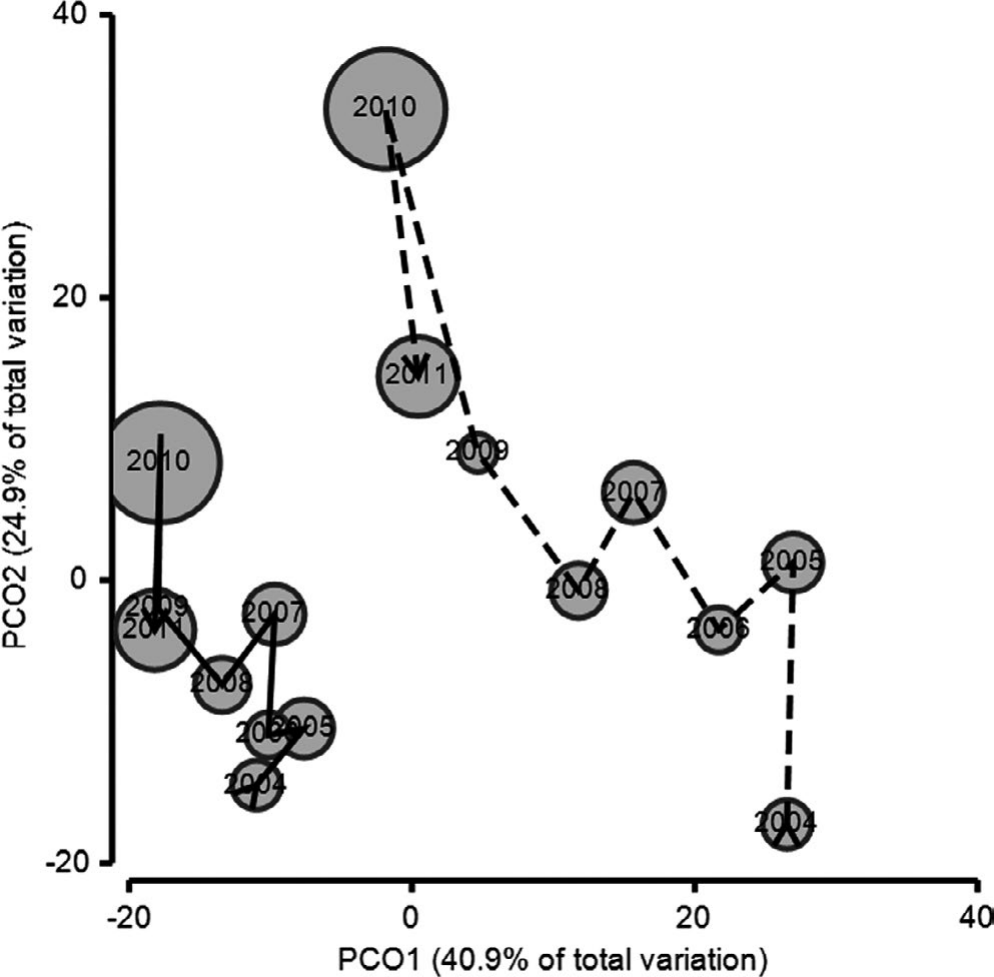
Temporal and spatial separation of bird assemblages at the Alice Springs Desert Park from 2004 to 2011 (solid line, irrigated area; dashed line, natural area), as shown by ordination with Principal Coordinates Analysis and trajectory overlay. The PCO1 axis represents 40.9% of variance and with irrigated and natural plots separated, and the PCO2 axis represents 24.9% of variance and correlates with temporal change. Data were plotted averaged by year and location to visualise the trajectory of overall change in irrigated and natural areas. Rainfall amounts have been overlayed in bubbles centred on year

The surveys produced more than 28,000 individual records of birds from 91 species. Of the 91 species, 28 were recorded in <10% of surveys and 13 in more than 90% of surveys. There was strong evidence of temporal change in bird communities among years (Pseudo‐*F*7, 440 = 3.9; *p* = .001) and seasons (Pseudo‐*F*3, 440 = 2.38; *p* = .001). Bird assemblages in the natural and irrigated areas also differed significantly (Pseudo‐*F*1, 440 = 2.01; *p* = .038), as did the interaction between year and irrigation (Pseudo‐*F*7, 440 = 1.48; *p* = .013), suggesting that temporal patterns differed in irrigated and nonirrigated plots. Figure 2 illustrates changes in composition of the bird community in the natural and irrigated areas, averaged across plots within irrigated and natural areas and samples within years, in relation to annual rainfall. The trajectories separate along the first PCO axis which explains approximately 40% of the total variation among communities. This axis appears to reflect overall differences in bird communities within irrigated and nonirrigated areas, and there was less variability through time in irrigated plots in relation to this axis. The second PCO axis appears to correspond primarily to temporal change associated with rainfall, and the similarities between the two trajectories in the relative position of different years along the axis suggest that changes in bird assemblages over time were similar in trend, but not as pronounced in the irrigated area as in the natural area. This is best illustrated when considering the year with the highest rainfall—2010—when 983 mm was recorded at the Park's weather station, more than three times the annual mean rainfall of 286 mm. Correspondingly, this year appears as an outlier with respect to the second PCO axis in both irrigated and natural areas in Figure 2, reflecting a considerable and consistent change in the composition of the bird community in this year. However, the change was less extreme at irrigated plots.

Dissimilarity between annual counts in the irrigated area ranged from 16.3% to 34.6% (mean 22.8%) in the SIMPER analysis, whilst those for the natural area ranged from 16.4% to 56.3% (mean 32.8%). These results, together with the reduced separation between years in irrigated compared to natural areas in Figure 2, indicate that bird assemblages were more stable across years in the irrigated area.

**FIGURE 3:**
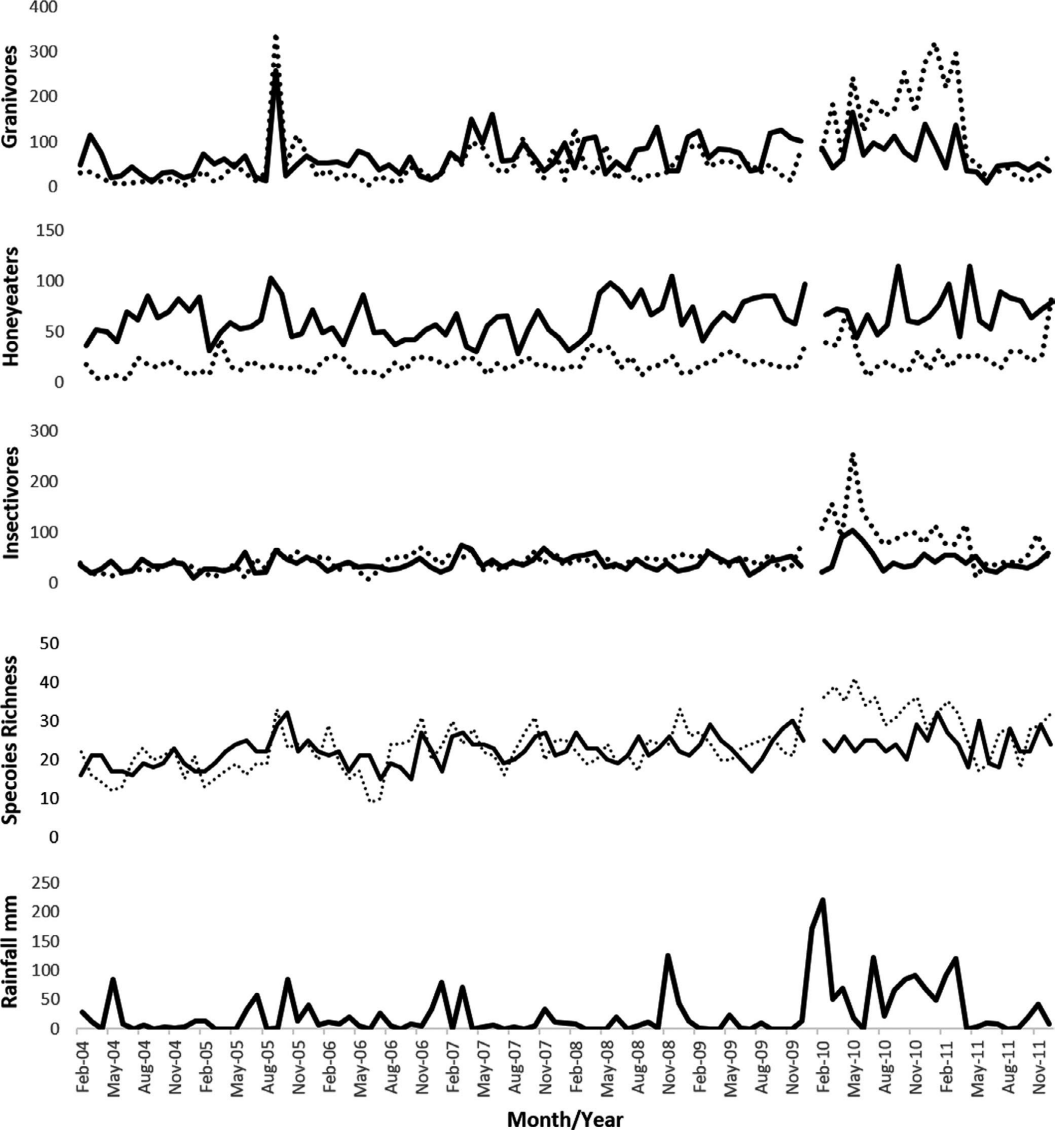
Total observations of the most abundant functional groups—granivores, honeyeaters and insectivores—and total species richness recorded in irrigated (solid lines) and natural areas (dotted lines); and rainfall per month from 2004 to 2011 at the Alice Springs Desert Park

**FIGURE 4:**
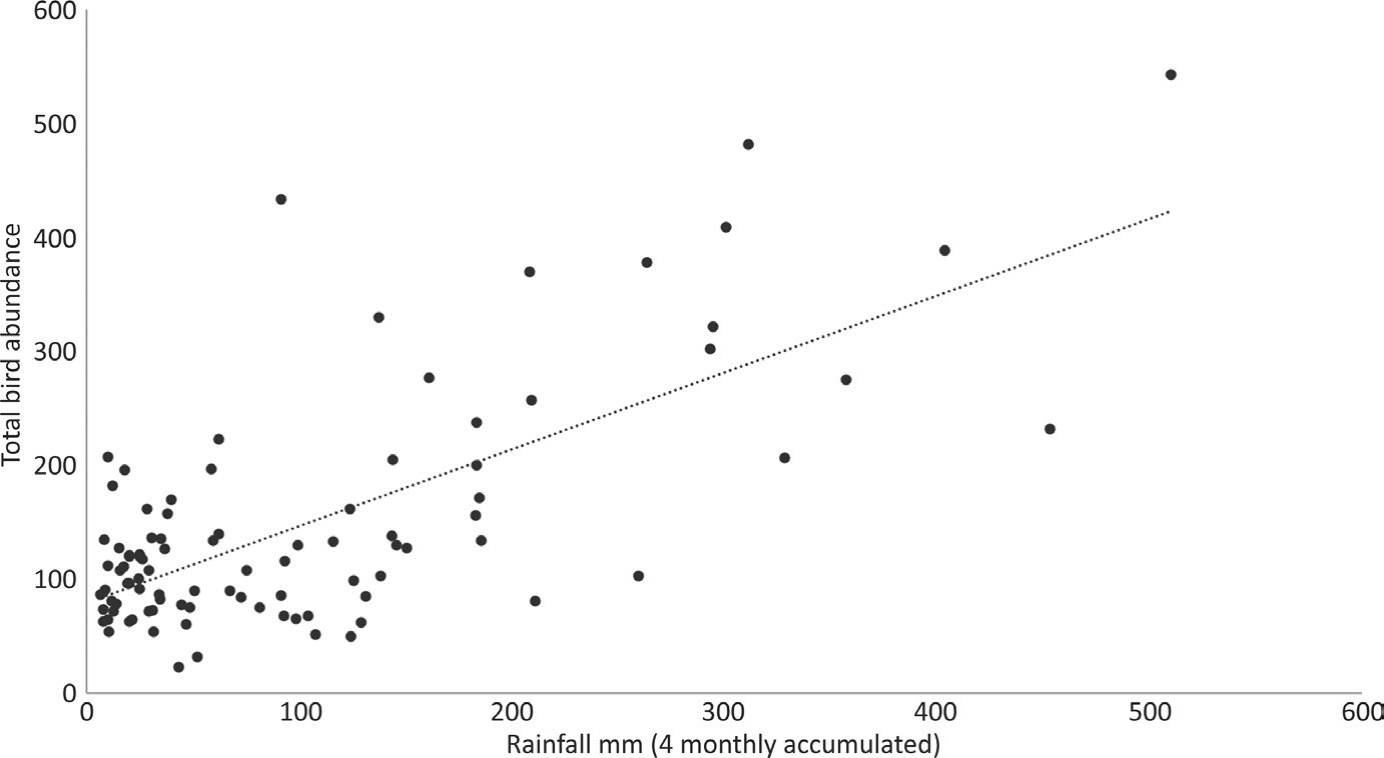
Relationship between bird abundance in the natural area of the Alice Springs Desert Park and rainfall accumulated in the 4 months prior to surveys

Differences in abundance between irrigated and natural plots and changes in abundance associated with high rainfall periods were investigated by separating birds into broad functional groups based on diet (Figure 3). Raptors and frugivorous birds were not investigated separately due to low numbers of individuals in these groups (they are included in the plot of species richness). Honeyeaters were the only group consistently more abundant in irrigated areas. Insectivorous and granivorous species tended to have similar abundances in irrigated and natural areas for the first 6 years of the survey but had higher abundance in natural areas from early 2010 up till mid‐2011, corresponding with unusually high rainfall (Figure 4). The abundance of granivores in natural plots also increased relative to irrigated plots at other times associated with smaller rainfall events, although the magnitude of this response was modest. The spike in abundance at both irrigated and nonirrigated areas in 2005 was due to a flock of 500–1,000 Budgerigars, *Melopsittacus undulatus*, which were active during surveys coinciding with high winter and spring rainfall in the area that year. Species richness tended to be slightly lower in the natural areas at most times except during 2010 and 2011, when this trend was reversed. Species richness was notably higher in natural areas in 2010, and the highest recorded at any time. Numbers of species counted each year in the irrigated and natural areas are listed in Table S1.

Our subsequent analyses examined patterns in natural plots, where productivity was not buffered by irrigation, in more detail. There was a significant positive correlation between total bird abundance in the natural area and cumulative rainfall over 1, 2, 3, 4, 5, and 6 months, the strongest correlation being with accumulated rainfall from the previous 4 months (Table 2; Figure 4). The abundance of both nomadic and sedentary species (Pascoe et al., 2019; Pavey & Nano, 2009) was significantly correlated with cumulative rainfall. Similarly, abundance of each of the three feeding guilds—granivores, insectivores, and nectarivores—was significantly positively correlated with rainfall although this was slightly weaker for honeyeaters (Table 2).

**TABLE 2:** Pearson's correlation coefficients between bird abundance in the natural area of the Alice Springs Desert Park and rainfall amounts accumulated over previous months; *p* values in brackets

Months of accumulated rainfall	Bird abundance	Nomadic species	Sedentary species	Granivores	Insectivores	Honeyeaters
1	0.534 (.001)	0.411 (.001)	0.470 (.001)	0.447 (.001)	0.469 (.001)	0.232 (.024)
2	0.599 (.001)	0.496 (.001)	0.498 (.001)	0.526 (.001)	0.503 (.001)	0.229 (.026)
3	0.669 (.001)	0.568 (.001)	0.553 (.001)	0.584 (.001)	0.571 (.001)	0.271 (.008)
4	0.684 (.001)	0.599 (.001)	0.556 (.001)	0.596 (.001)	0.592 (.001)	0.298 (.003)
5	0.673 (.001)	0.583 (.001)	0.563 (.001)	0.603 (.001)	0.570 (.001)	0.305 (.003)
6	0.643 (.001)	0.514 (.001)	0.588 (.001)	0.597 (.001)	0.530 (.001)	0.271 (.008)

### 3.2 Temporal change in bird assemblages in relation to season in irrigated and natural areas

**FIGURE 5:**
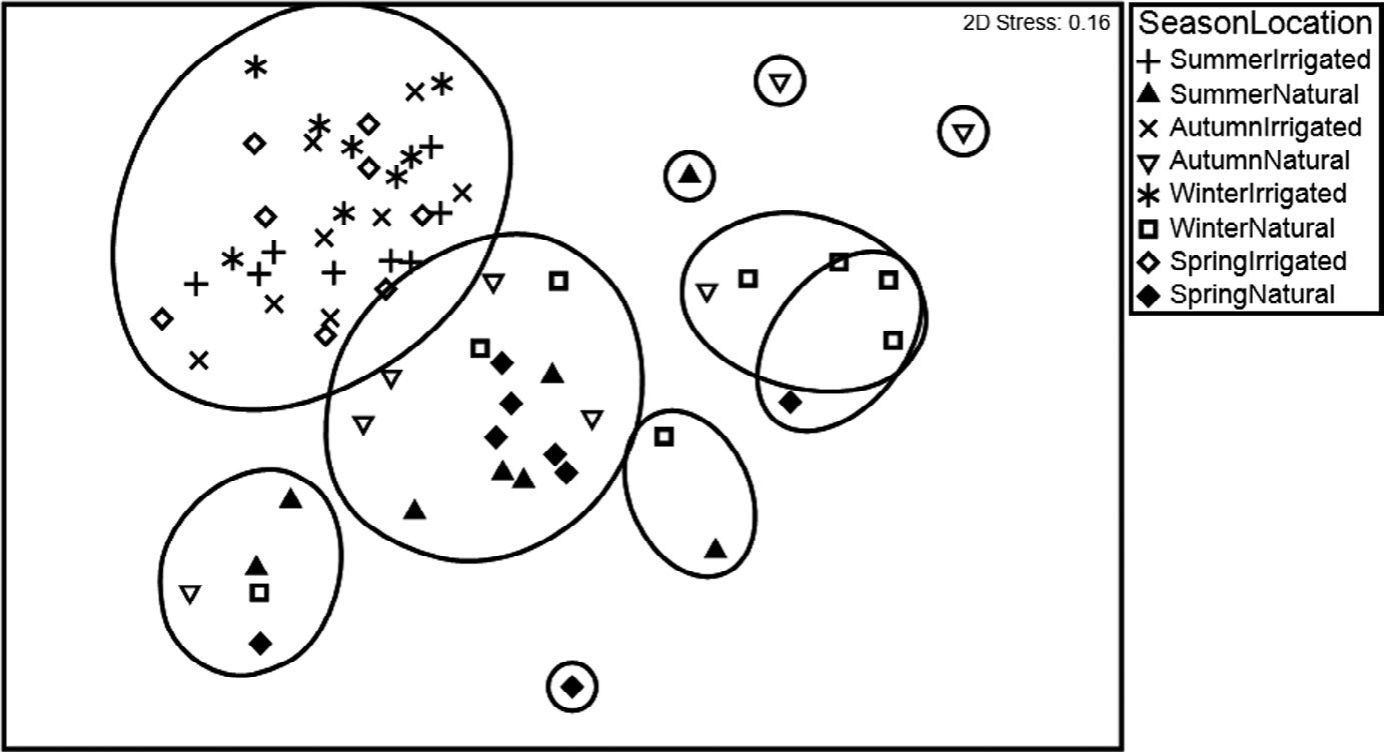
Ordination plot representing similarities between bird assemblages in irrigated and natural plots in different seasons. Data for each season and plot were pooled across 8 years. Overlay of hierarchical cluster analysis overlay on the nMDS plot indicates 60% similarity

In addition to temporal change associated with rainfall, we also predicted that birds would show seasonal shifts, such as migrants arriving in spring from northern Australia. Initially, we analyzed all data in irrigated and nonirrigated plots together. Figure 5 shows that bird assemblages (represented in a 2‐dimensional ordination) at irrigated plots were distinct from those in nonirrigated areas and did not seem to vary seasonally. This clumping of irrigated plots in the ordination represents a masking or buffering of ephemeral fluctuations compared to natural areas. Bird assemblages in natural plots were more variable with some weak clustering of samples from winter, spring, and summer.

**FIGURE 6:**
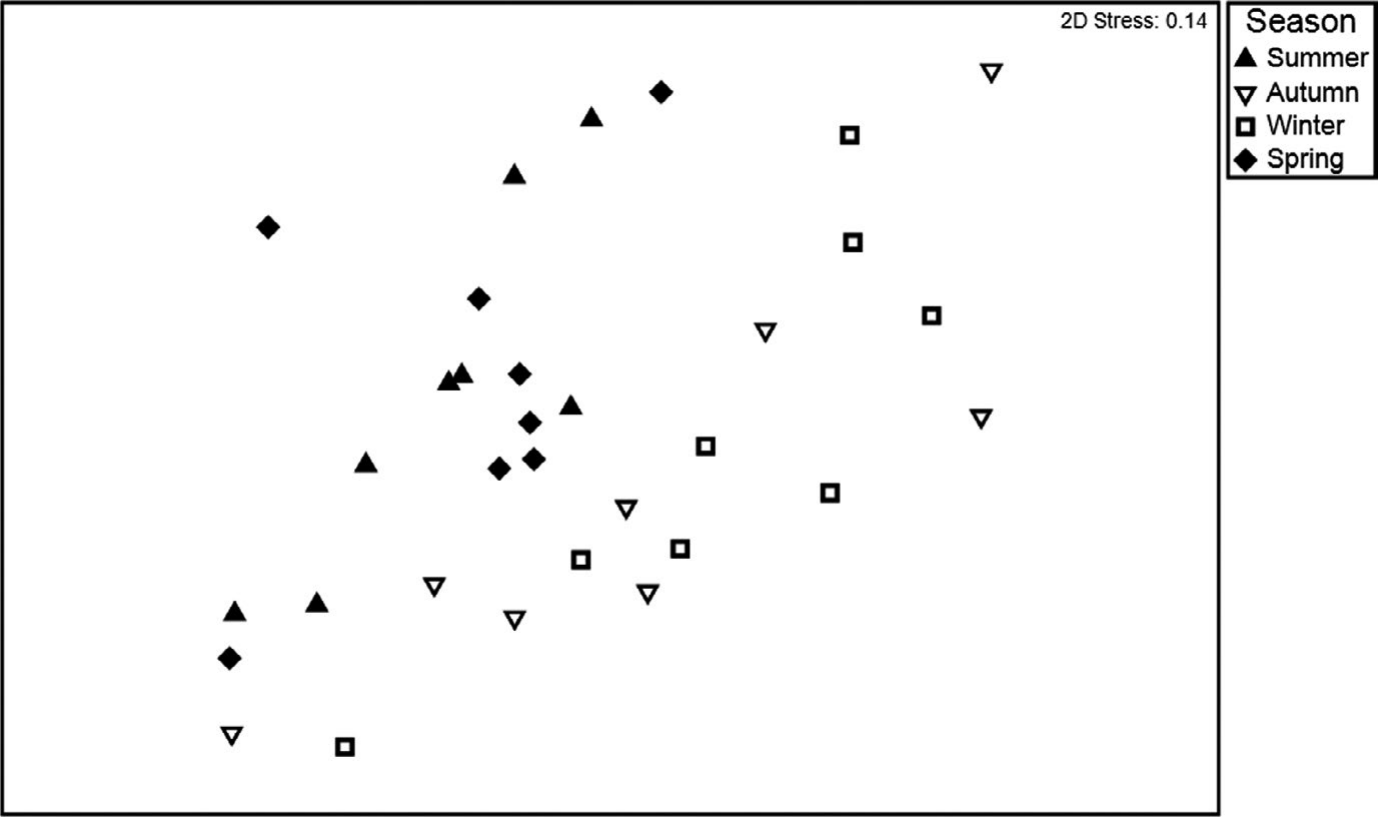
Similarities in composition of bird assemblages in natural areas of the Alice Springs Desert Park according to season represented as an nMDS plot. Data for each season and plot were pooled across 8 years

To explore seasonal effects in the natural area further, data from the irrigated area were excluded, and a separate Bray–Curtis similarity matrix was generated. The resulting nMDS plot shows separation between spring/summer and autumn/winter, suggesting that distinct bird assemblages were present in natural areas at these times (Figure 6). Indeed, season was a significant factor explaining bird communities in the natural area (Pseudo‐*F*3, 220 = 2.11; *p* = .001). Pairwise tests indicated significant differences between winter and summer (*p* < .005), winter and spring (*p* < .006) and autumn and spring (*p* < .01). Assemblages did not differ significantly between spring and summer, or between autumn and winter; there were no significant differences between autumn and summer, although points representing these seasons appear to separate in the nMDS plot.

### 3.3 Spatial differentiation among bird assemblages

**FIGURE 7:**
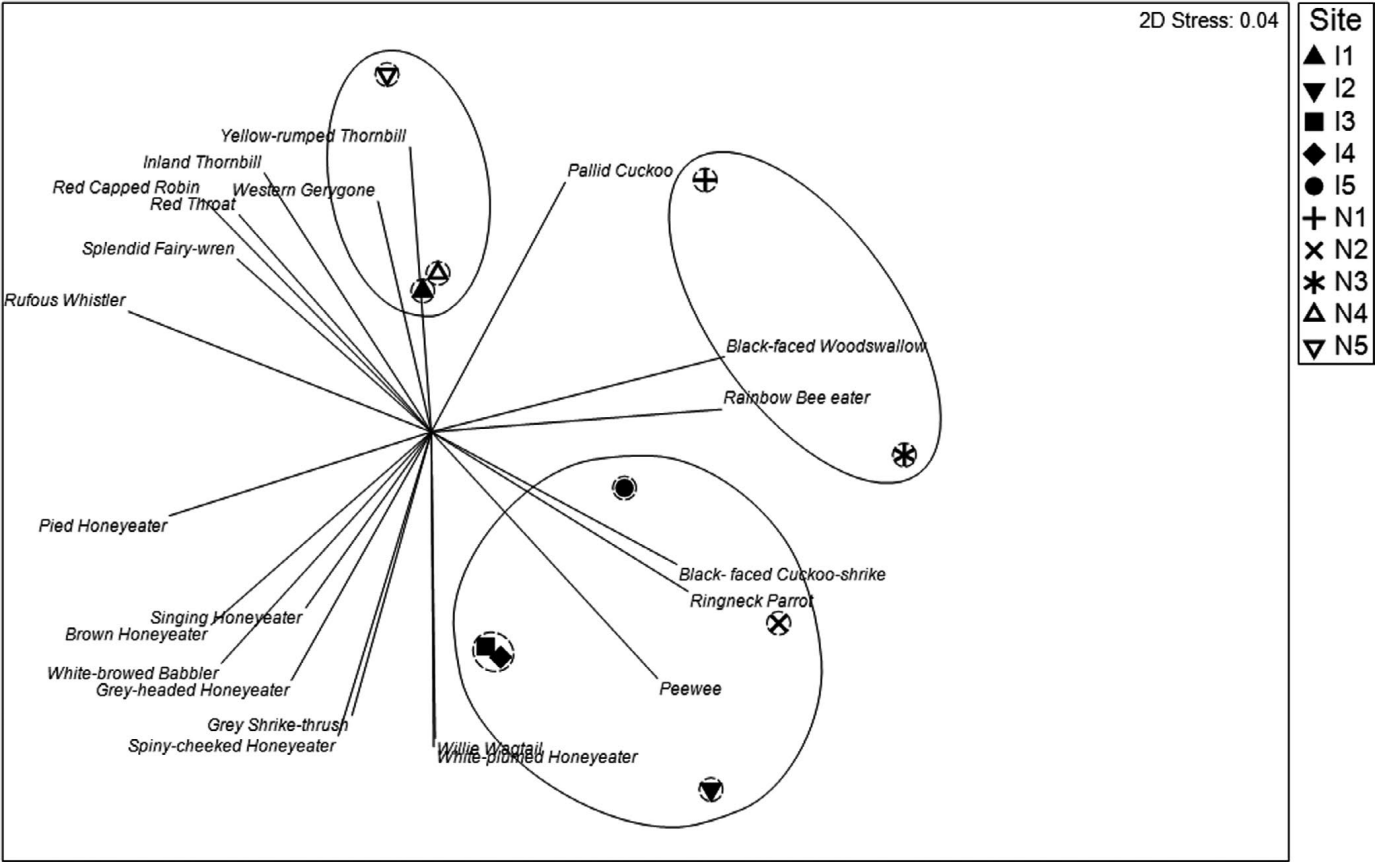
Spatial differentiation in composition of bird assemblages in irrigated (I) and natural (N) areas of the Alice Springs Desert Park, as shown by hierarchical cluster analysis, grouped at 60% (solid line) and 80% (dashed line) similarity in composition; vectors have been overlaid indicating contributions of selected individual species (Pearson correlation > 0.8) to the positioning of plots. Data from all surveys were pooled into one measure for each plot prior to analysis

To explore the impact of habitat differences among plots on the bird assemblages we calculated mean counts of each species per plot per year, generated a similarity matrix, and tested for differences between plots with PERMANOVA, with Year, Location, Season as fixed factors and Plot and Month as random. Differences between plots were significant (Pseudo‐*F*8, 440 = 32.9; *p* = .001) as were interactions between plots and years (Pseudo‐*F*_56, 440_ = 2.34; *p* = .001) and plots and seasons (Pseudo‐*F*_24, 440_ = 1.79; *p* = .001). Plot differences were explored further by pooling data for each plot across the entire survey period and overlaying a cluster analysis showing 60% similarity on the nMDS ordination of bird assemblages at plots (Figure 7). Although there was clear evidence of similarity in bird assemblages among four of the five irrigated plots the similarity of assemblages between the fifth plot I1 with natural plots N4 and N5 is noteworthy. The clustering of these plots and some of the other patterns evident from Figure 7 appear to correspond to vegetation assemblages at the survey plots. Plots I1, N4, and N5 were all dominated by *A. aneura* and *A. kempeana* (Table 1). The species overlay indicates that a number of species influencing this grouping (Red‐capped Robin, *Petroica goodenovii*, Rufous Whistler, *Pachycephala rufiventris*, Inland Thornbill, *Acanthiza apicalis* and Splendid Fairy‐ Wren, *Malurus splendens*) are core mulga species as classified by Cody (1994). Another group of mainly irrigated plots with relatively high similarity included N2, a natural plot just outside the irrigated area with a creek‐line with *Eucalyptus camaldulensis* and bordered on gardens of planted eucalypts; large honeyeater species were primarily associated with these plots. The relative similarity between I2, I3, I4, and I5, and N2 is also at least partially accountable by their close location to each other (Figure 1). Finally, N1 and N3 had relatively similar assemblages and these plots were characterized by open vegetation, N1 with *Corymbia opaca* and some *Acacia* shruband, and N3 a creek‐line with some *eucalypts*, *Acacia estrophiolata* and *Acacia victoriae*. Species associated with these relatively open habitats of this grouping were Crimson Chat, *Epthianura tricolor*, and Rainbow Bee‐eater, *Merops ornatus*. Species counts for each plot for the entire survey period are listed in Table S2.

**FIGURE 8:**
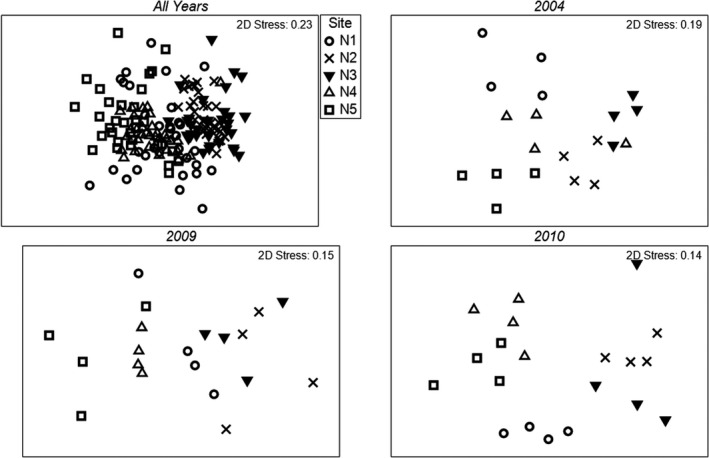
Composition of bird assemblages averaged by season and plot in the natural area of the Alice Springs Desert Park, based on nMDS ordinations of combined data showing plot differences for: all years of the survey; 2008 (a year closest to the recorded median rainfall); 2009 (the driest year); and 2010 (the wettest year)

These results suggest that in some instances underlying differences in vegetation were as important as irrigation or proximity in determining the composition of bird assemblages despite the strong influence of the latter factors. However, mindful of the partly artificial nature of the habitats within the Park, and the spatial proximity and nonindependence of irrigated plots, we excluded these plots and focused on the natural areas to further explore the interplay between temporal and spatial patterning. Our aim was to determine whether the distinctiveness of bird assemblages among natural plots remained consistent across time. The relative positioning of plots was examined on nMDS graphs generated from a separate Bray–Curtis similarity index, summarized by year and season. Graphs for all times, and in years representing low, intermediate, and high rainfall (Figure 8), show that samples within a plot tended to clump irrespective of year and season (see all years) and that bird assemblages at each plot were at least partially distinct from each other irrespective of season or rainfall conditions (see patterns for 2008, 2009, and 2010 which represent an average year and the driest and wettest years, respectively). These results suggest that habitat or vegetation structure is influential in determining composition of avian assemblages, even when assemblages are highly variable through time.

## 4 DISCUSSION

### 4.1 Rainfall and temperature are strong drivers of temporal change in the avifauna in natural areas

This study examined change in avian assemblages in relation to rainfall, but also considering seasonal changes. Comparing the responses of birds to rainfall in natural areas with those in a physically similar irrigated area of the Alice Springs Desert Park, where productivity remained permanently enhanced, provided a unique opportunity to distinguish effects of rainfall‐related productivity from other temporal changes. The 8‐year survey duration included a period of “normal” low rainfall as well as a period of extremely high precipitation. The most pronounced changes in the composition, abundance, and species richness of birds were observed in 2010, coinciding with high rainfall throughout arid Australia (Wardle et al., 2013). Temporal change in bird assemblages in response to this event—namely increases in abundance of insectivores and granivores, increase in species richness and changes in composition—was markedly greater in natural than irrigated areas, which is consistent with our prediction that continuous production in the irrigated area has a buffering effect on bird assemblages. Research conducted by Doucette et al. (2012) found that the biomass of terrestrial and arboreal arthropods was consistently higher in irrigated areas of the Desert Park compared to similar nonirrigated areas elsewhere in the MacDonnell Ranges and supports the notion that irrigation, together with supplemental planting, directly enhanced the plant and invertebrate food resources on which many birds depend, although these resources were still tied to seasonal patterns in the life cycles of plants and invertebrates. The changes in abundance of birds at the natural plots corresponded directly with rainfall patterns over a decadal timeframe and, especially when contrasted with the greater stability observed within the irrigated area, is strong support for the proposition that abundance of many bird species in arid Australia is limited by rainfall‐driven fluctuations in the availability of food resources.

One of the most dramatic changes in avian composition was a large influx of granivores (Budgerigars, Zebra Finches, *Taeniopygia guttata*, and Diamond Doves, *Geopelia cuneata*) at natural plots in 2010. A distinct spike in the abundance of granivores was also obvious in 2005, when many Budgerigars were recorded across both natural and irrigated areas. The presence of Budgerigar flocks likely corresponded to regionally successful breeding by this species after winter rain and follow‐up spring rain produced abundant seeding grasses. These patterns reaffirm that rainfall is a strong driver of temporal change in the composition of avian granivore communities, for example, see Tischler et al. (2013). The substantial increase in abundance of insectivores in natural plots relative to irrigated plots during the high rainfall period reflected enhanced breeding of resident species such as Splendid Fairy‐Wrens, *Malurus splendens*, as well as an influx of more mobile species such as Crimson Chats, *Epthianura tricolor*, White‐winged Trillers, *Lalage sueurii*, and Masked Woodswallows, *Artamus personatus*, and Black‐faced Woodswallows, *Artamus cinereus*) (Table S1). Comparisons with irrigated areas provide additional insights into the important factors structuring bird composition and abundance.

Whilst populations of granivores and insectivores fluctuated with changing rainfall and resultant primary productivity, especially at natural plots, populations of honeyeaters at both irrigated and natural plots remained relatively stable throughout the survey period. Irrigated plots supported a greater abundance of honeyeaters at almost all times. Sustained primary productivity enabled by irrigation ensured that flowering of plants was maintained at high levels and, indirectly, a high biomass of invertebrates was maintained. The enhancement of these resources, together with the habitats represented (some of which included substantial plantings of flowering species), account for the abundance and stability of honeyeaters at irrigated plots. Two honeyeater species dominated the assemblage. White‐plumed Honeyeaters, *Lichenostomus penicillatus*, are eucalypt specialists dominant in areas of high productivity (Barrett et al., 2008); they were the principal species at plots with abundant River Red Gums and in other created habitats with a variety of eucalypts. Spiny‐cheeked Honeyeaters, *Acanthagenys rufogularis*, were also abundant throughout the irrigated area. The natural survey plots did not include extensive areas of eucalypts or plants such as *Eremophila* and *Grevillea*, known to attract honeyeaters when in flower. The relatively modest increase in abundance of honeyeaters in response to rainfall, especially at natural plots, suggests primary productivity may not be the most important factor limiting honeyeater abundances. The relative contributions of nectar and insects to the diets of arid‐zone honeyeaters shifts markedly over time depending on the availability of nectar, but the presence of suitable flowers is an important factor determining their distribution, particularly for mobile species. It is likely that the relatively low abundance of nectar‐producing plants in natural areas limited the abundance of honeyeaters, even during high rainfall periods. In other words, habitat may have been the most important factor governing abundance and distribution of this group.

In contrast to honeyeaters, abundance of insectivores and granivores was not enhanced at irrigated plots in dry times. This is a surprising result considering that invertebrate abundance is higher and more stable at such sites (Doucette et al., 2012). As well as not being elevated in dry times, the abundance of insectivores and granivores also remained relatively stable at irrigated plots during the boom period, when large increases occurred at natural plots. This contrasting pattern suggests that the abundance of these groups was limited by rainfall‐related productivity at the natural plots but by other factors at irrigated ones. We suggest that inter and intraspecific competition may have limited further population increases in irrigated plots across all foraging guilds during wet times, despite some further enhancement of food resources at those times. As the abundance of honeyeaters in irrigated plots was the hallmark of these areas, it is possible that granivorous and insectivorous species were limited to some extent by aggressive (e.g., chasing) and competitive interactions with dominant honeyeaters.

The composition and abundance of bird assemblages in the irrigated and especially the natural areas were greatly influenced by rainfall, but seasonal change was also evident at natural plots, with bird assemblages in spring and summer differentiated from those in autumn and winter. The predominant cause of seasonal change was summer migrants, such as Rainbow Bee‐eaters and, apart from the year of heavy rainfall of 2010, appeared to be consistent between years. Seasonal changes were less evident in irrigated areas. We suggest that this discrepancy stems from the habit of seasonal migrants of seeking distinctive habitat features for nesting and foraging. For example, Rainbow Bee‐eaters were more abundant at N3 (216 observations) than at any other plot, less so at plots N2, N1, and I6 (78, 45, 14 observations, respectively), and were observed lesser than a total of 10 times at other plots. The plots where they were abundant featured low rises and drainage line banks, used for burrowing and nesting, and open areas with high perches suitable for hunting. We propose that the local distribution of Rainbow Bee‐eaters and other seasonal migrants coincide with favorable breeding sites and is less responsive to local‐scale variation in food resources, in comparison with sedentary and nomadic species.

### 4.2 Bird assemblages are spatially distinctive at the plot scale

The relative proximity and lack of spatial independence among irrigated plots is likely to account, at least in part, for the similarity in bird assemblages among some plots. For example, I3 and I4 are adjacent to each other and had similar bird assemblages but different plant communities. We also acknowledge that differences between irrigated and natural areas, including the presence of buffelgrass at natural plots and exclusion of some predators from irrigated areas, potentially reduced relative abundance and richness of birds at natural plots (e.g., see Paltridge, 2002; Young & Schlesinger, 2015) or otherwise contributed to similarities among irrigated and nonirrigated plots respectively. There is currently only limited evidence of the impacts of these factors on central Australian avifauna; none‐the‐less, if such effects existed, they cannot confidently be separated from the effects of irrigation because of limitations to the survey design, so we remain cautious in drawing conclusions about causes of spatial differences. However, the similarity of assemblages at plots I1 and N4, both mulga shrubland, despite the distance between them and the fact that one was irrigated, is evidence of the importance of habitat in determining bird assemblages. It also suggests that the assemblage of birds at each plot was distinctive, despite probable movement of some individuals among those plots that were close together. The spatial variation of bird assemblages among our plots is consistent with Pavey and Nano (2009), and a variety of studies from mesic environments (Arnold, 1988), in suggesting that that habitat structure underpins the composition of avian assemblages, on which the effects of rainfall are superimposed to varying degrees. In our case, natural plots tended to be more open and grassier and may have provided more opportunities for granivorous and insectivorous species during boom periods. Conversely, we noted that greater numbers of nectar‐producing plants at irrigated plots (in combination with continuous production) may have allowed colonial honeyeaters to occur more regularly. We conclude that spatial and temporal differences among bird assemblages of the natural and irrigated areas had multiple causes including the underlying structure and composition of the vegetation and interspecific interactions—especially at irrigated plots, on which were imposed patterns of resource availability stimulated by rainfall and irrigation.

### 4.3 Conservation implications

As temperatures and the frequency of drought increase as projected under future climate change, the need for drought refuges where animals can access water or moist conditions is likely to become increasingly important. It behoves us to briefly consider the potential role of artificially irrigated areas in meeting this need. Indeed, the generally more stable abundances that were recorded at irrigated plots suggest the resistance of birds to drought was enhanced in these areas. However, there was no clear evidence that that the resilience of species to drought (i.e., the ability for populations to bounce back following the onset of mesic conditions) was enhanced, in fact, the positive response to mesic conditions was greater in natural areas. Although, as already discussed, this can partly be explained by habitat differences and interspecific interactions, it is none‐the‐less broadly consistent with other research on natural Australian habitats that represent hydric refugia (Selwood et al., 2015) and studies that have investigated relationships between fauna and productivity gradients in other arid regions worldwide. In Africa increased rainfall (due to seasonal fluctuations and geographical rainfall gradients) is associated with increased richness of bird species but, conversely, avian functional diversity is decreased in these more productive areas (Seymour et al., 2015). Seymour et al. (2015) suggest that although enhanced productivity may reduce competition among functionally similar species, leading to an increase in their abundance and richness, it also favors stronger competitors. In contrast, higher disturbance, such as greater exposure to drought, creates opportunities for other less‐competitive species and prevents their exclusion by more dominant species.

The strategies by which different bird species survive low resource periods fall within a spectrum from resistance (i.e., species that maintain relatively stable abundances irrespective of temporal fluctuations in resources) to resilience (species that disappear locally or decline dramatically in dry times but quickly return/recover following high rainfall). The former are often relatively sedentary residents of an area with somewhat flexible diets and the later more likely to be nomads, often with more specialized diets (Pascoe et al., 2019). Artificial—and natural—hydric refugia may be more likely to benefit those that rely on resistance rather than resilience strategies to survive drought. Benefits could therefore be largely limited to a group of similar species resident in the local area. Dedicated research is required to better understand the extent to which habitats with comparatively high water and nutrient availability— such as regional and remote townships— are used as drought refuge by wide‐ranging and eruptive species in arid environments and their potential, thereby, to facilitate repopulation in the wider landscape when the drought breaks.

## 5 CONCLUSION

We used a novel approach to test hypotheses about the drivers of temporal change in bird assemblages in arid Australia: we compared temporal changes in bird assemblages in a natural area with those in an area where fluctuations in productivity were smoothed out by irrigation and supplemental planting. This contrast, together with the length of our study, enabled us to demonstrate that the abundance of insectivorous and granivorous birds in natural areas is limited during dry periods by reduced availability of resources; when resource limitation was mitigated by irrigation, avian assemblages fluctuated less. Irrespective of temporal change in resources, however, distinctive bird assemblages were present in different habitats, supporting previous conclusions that species of the region are constrained by fixed habitat parameters governing their ability to forage and breed (Cody, 1994; Pavey & Nano, 2009). Our study adds to the body of knowledge that is gradually accumulating about the complex spatial and temporal dynamics of bird communities in arid regions. Understanding how fauna responds to extreme temporal variability is particularly important as climate change further exacerbates rainfall variability worldwide and conservation interventions to enhance resilience and resistance of species to extreme conditions are more widely considered.

## CONFLICT OF INTEREST

The authors declare no conflict of interest.

## AUTHOR CONTRIBUTION

**Bruce A. Pascoe:** Conceptualization (lead); Data curation (lead); Formal analysis (lead); Investigation (lead); Project administration (equal); Writing‐original draft (lead); Writing‐review & editing (equal). **Chris R. Pavey:** Conceptualization (equal); Investigation (supporting); Supervision (supporting); Writing‐review & editing (supporting). **Stephen R. Morton:** Conceptualization (supporting); Supervision (supporting); Writing‐review & editing (supporting). **Christine A. Schlesinger:** Conceptualization (equal); Formal analysis (supporting); Project administration (equal); Supervision (lead); Writing‐review & editing (equal).

## Supporting information

Table S1‐S2

## Data Availability

Avian survey data are available on Dryad at https://doi.org/10.5061/dryad.51c59zw7f
