# Supplementary material for: Dynamics of bird assemblages in response to temporally and spatially variable resources in arid Australia
Source: Ecol Evol. 2021 Mar 17;11(9):3977–90. doi: 10.1002/ece3.7293 (PMC8093688; doi:10.1002/ece3.7293)
Supplement: Supplementary file 1 — Table S1‐S2 [file ECE3-11-3977-s001.docx]

Table S1. Annual species counts in irrigated (I) and natural (N - shaded) areas for survey period.

|  | 2004 | | 2005 | | 2006 | | 2007 | | 2008 | | 2009 | | 2010 | | 2011 | |
| --- | --- | --- | --- | --- | --- | --- | --- | --- | --- | --- | --- | --- | --- | --- | --- | --- |
|  | I | N | I | N | I | N | I | N | I | N | I | N | I | N | I | N |
| *Coturnix pectoralis* | 0 | 0 | 0 | 0 | 0 | 0 | 0 | 0 | 0 | 0 | 0 | 0 | 0 | 0 | 0 | 1 |
| *Coturnix ypsilophora* | 0 | 0 | 0 | 0 | 0 | 0 | 0 | 0 | 0 | 0 | 0 | 0 | 0 | 0 | 10 | 2 |
| *Aquila audax* | 0 | 0 | 0 | 2 | 0 | 0 | 1 | 0 | 0 | 0 | 0 | 1 | 0 | 0 | 0 | 0 |
| *Elanus axillaris* | 0 | 0 | 0 | 0 | 0 | 0 | 0 | 0 | 0 | 0 | 0 | 0 | 0 | 10 | 0 | 12 |
| *Milvus migrans* | 0 | 1 | 1 | 3 | 3 | 9 | 4 | 4 | 5 | 4 | 0 | 10 | 0 | 6 | 2 | 4 |
| *Haliastur sphenurus* | 0 | 0 | 0 | 0 | 0 | 0 | 0 | 0 | 0 | 2 | 1 | 0 | 4 | 2 | 3 | 0 |
| *Accipter fasciatus* | 0 | 0 | 4 | 1 | 4 | 1 | 1 | 1 | 1 | 1 | 8 | 4 | 0 | 4 | 1 | 3 |
| *Accipter cirrocephalus* | 2 | 0 | 1 | 1 | 0 | 0 | 0 | 1 | 0 | 0 | 0 | 0 | 0 | 1 | 3 | 1 |
| *Falco subneger* | 0 | 1 | 0 | 1 | 0 | 0 | 0 | 0 | 0 | 0 | 0 | 1 | 0 | 1 | 0 | 2 |
| *Falco berigora* | 1 | 1 | 3 | 6 | 0 | 0 | 4 | 2 | 0 | 5 | 0 | 2 | 1 | 10 | 1 | 6 |
| *Falco cenchroides* | 0 | 5 | 2 | 2 | 2 | 0 | 1 | 1 | 1 | 1 | 0 | 0 | 1 | 0 | 0 | 1 |
| *Falco peregrinus* | 0 | 0 | 0 | 0 | 0 | 0 | 0 | 0 | 1 | 0 | 0 | 0 | 0 | 0 | 0 | 0 |
| *Falco longipennis* | 0 | 0 | 0 | 0 | 0 | 0 | 1 | 1 | 0 | 0 | 0 | 0 | 0 | 0 | 0 | 1 |
| *Turnix velox* | 0 | 0 | 0 | 0 | 0 | 0 | 0 | 2 | 0 | 2 | 0 | 1 | 4 | 43 | 4 | 2 |
| *Burhinus grallarius* | 0 | 0 | 0 | 0 | 4 | 0 | 1 | 0 | 0 | 0 | 10 | 0 | 9 | 0 | 34 | 0 |
| *Geopelia placida* | 1 | 0 | 7 | 2 | 4 | 0 | 10 | 2 | 3 | 3 | 2 | 4 | 9 | 0 | 2 | 1 |
| *Geopelia cuneata* | 1 | 1 | 53 | 8 | 1 | 0 | 27 | 17 | 18 | 16 | 29 | 58 | 182 | 361 | 54 | 189 |
| *Phaps chalcoptera* | 0 | 1 | 5 | 4 | 3 | 2 | 2 | 1 | 15 | 3 | 11 | 4 | 2 | 2 | 1 | 2 |
| *Ochyphaps lophotes* | 70 | 19 | 86 | 12 | 198 | 86 | 247 | 31 | 135 | 80 | 183 | 66 | 236 | 118 | 247 | 174 |
| *Geophaps plumifera* | 0 | 0 | 0 | 1 | 0 | 0 | 0 | 1 | 1 | 0 | 2 | 0 | 0 | 3 | 1 | 2 |
| *Podargus strigoides* | 0 | 1 | 0 | 0 | 0 | 0 | 0 | 0 | 0 | 0 | 0 | 0 | 0 | 0 | 0 | 0 |
| *Eurostopodus argus* | 4 | 1 | 0 | 0 | 0 | 1 | 0 | 0 | 0 | 0 | 0 | 1 | 0 | 0 | 0 | 0 |
| *Calyptorhynchus banksii* | 0 | 0 | 2 | 0 | 0 | 0 | 0 | 0 | 0 | 0 | 0 | 0 | 0 | 11 | 0 | 2 |
| *Eolophus roseicapilla* | 4 | 37 | 82 | 103 | 67 | 25 | 47 | 21 | 166 | 36 | 87 | 71 | 69 | 49 | 11 | 133 |
| *Cacatua sanguinea* | 0 | 0 | 0 | 0 | 0 | 0 | 0 | 0 | 0 | 0 | 0 | 0 | 0 | 0 | 0 | 2 |
| *Nymphicus hollandicus* | 0 | 0 | 0 | 0 | 0 | 0 | 0 | 0 | 0 | 0 | 0 | 0 | 5 | 8 | 0 | 0 |
| *Melopsittacus undulatus* | 33 | 3 | 253 | 492 | 35 | 11 | 207 | 278 | 15 | 65 | 172 | 117 | 185 | 337 | 70 | 191 |
| *Barnadius zonarius* | 58 | 89 | 71 | 97 | 55 | 76 | 84 | 67 | 68 | 59 | 71 | 59 | 73 | 100 | 107 | 125 |
| *Psephotus varius* | 4 | 0 | 2 | 0 | 5 | 0 | 7 | 0 | 4 | 0 | 0 | 2 | 0 | 0 | 0 | 4 |
| *Cacomantis pallidus* | 0 | 0 | 0 | 1 | 0 | 2 | 0 | 0 | 0 | 2 | 0 | 2 | 0 | 5 | 0 | 3 |
| *Chalcites osculans* | 0 | 0 | 3 | 0 | 0 | 0 | 0 | 0 | 0 | 1 | 0 | 3 | 0 | 1 | 0 | 0 |
| *Chalcites basalis* | 0 | 0 | 3 | 1 | 3 | 1 | 1 | 2 | 1 | 3 | 3 | 1 | 1 | 10 | 1 | 2 |
| *Scythrops novaehollandiae* | 0 | 0 | 0 | 0 | 2 | 0 | 0 | 0 | 0 | 0 | 0 | 0 | 0 | 0 | 0 | 0 |
| *Todiramphus pyrrhopygius* | 0 | 8 | 4 | 10 | 1 | 24 | 3 | 32 | 0 | 12 | 1 | 14 | 7 | 36 | 10 | 20 |
| *Todiramphus sanctus* | 0 | 0 | 1 | 0 | 0 | 2 | 0 | 1 | 0 | 0 | 0 | 5 | 0 | 1 | 2 | 1 |
| *Merops ornatus* | 0 | 14 | 4 | 33 | 2 | 45 | 5 | 43 | 5 | 43 | 11 | 63 | 1 | 37 | 2 | 67 |
| *Malurus splendens* | 84 | 53 | 61 | 65 | 138 | 140 | 135 | 151 | 137 | 125 | 122 | 128 | 61 | 164 | 115 | 170 |
| *Malurus lamberti* | 24 | 39 | 29 | 9 | 14 | 10 | 24 | 17 | 17 | 22 | 9 | 6 | 6 | 8 | 7 | 21 |
| *Pardalotus rubricatus* | 2 | 3 | 1 | 0 | 0 | 1 | 0 | 3 | 0 | 3 | 0 | 3 | 0 | 3 | 0 | 0 |
| *Pardalotus striatus* | 2 | 0 | 0 | 0 | 0 | 0 | 1 | 0 | 0 | 0 | 0 | 1 | 0 | 5 | 0 | 0 |
| *Pyrrholaemus brunneus* | 0 | 0 | 0 | 0 | 0 | 0 | 0 | 0 | 1 | 0 | 4 | 0 | 0 | 8 | 2 | 4 |
| *Gerygone fusca* | 9 | 3 | 1 | 10 | 1 | 5 | 10 | 8 | 10 | 6 | 7 | 5 | 4 | 26 | 2 | 8 |
| *Smicromis brevirostris* | 5 | 0 | 50 | 8 | 23 | 4 | 25 | 8 | 32 | 14 | 9 | 2 | 11 | 12 | 0 | 7 |
| *Acanthiza chrysorrhoa* | 21 | 15 | 14 | 45 | 27 | 35 | 27 | 53 | 41 | 38 | 13 | 25 | 10 | 61 | 16 | 56 |
| *Acanthiza apicalis* | 12 | 13 | 22 | 54 | 6 | 34 | 15 | 39 | 33 | 71 | 14 | 41 | 3 | 22 | 5 | 36 |
| *Acanthiza uropygialis* | 0 | 0 | 7 | 12 | 10 | 29 | 7 | 22 | 2 | 9 | 1 | 0 | 0 | 6 | 0 | 3 |
| *Acanthiza robustirostris* | 0 | 0 | 0 | 0 | 0 | 0 | 0 | 0 | 2 | 4 | 5 | 3 | 0 | 0 | 0 | 0 |
| *Aphelocephala leucopsis* | 0 | 0 | 0 | 0 | 0 | 0 | 0 | 0 | 0 | 0 | 0 | 0 | 1 | 2 | 0 | 0 |
| *Acanthagenys rufogularis* | 228 | 53 | 243 | 72 | 203 | 87 | 187 | 63 | 288 | 97 | 340 | 107 | 180 | 56 | 240 | 148 |
| *Lichenostomus virescens* | 100 | 22 | 105 | 28 | 97 | 38 | 135 | 40 | 102 | 68 | 136 | 76 | 98 | 87 | 124 | 84 |
| *Lichenostomus keartlandi* | 5 | 1 | 12 | 10 | 9 | 2 | 3 | 1 | 18 | 6 | 18 | 6 | 17 | 2 | 29 | 8 |
| *Lichenostomus penicillatus* | 209 | 35 | 184 | 35 | 173 | 33 | 170 | 69 | 228 | 37 | 223 | 34 | 400 | 68 | 472 | 88 |
| *Lichmera indistincta* | 119 | 36 | 152 | 39 | 134 | 41 | 120 | 27 | 206 | 29 | 132 | 27 | 27 | 9 | 109 | 9 |
| *Pumella albifrons* | 0 | 0 | 3 | 0 | 1 | 0 | 0 | 5 | 4 | 3 | 0 | 0 | 0 | 0 | 4 | 0 |
| *Conopophila whitei* | 0 | 0 | 1 | 0 | 0 | 2 | 0 | 0 | 1 | 0 | 1 | 2 | 0 | 0 | 0 | 0 |
| *Sugomel nigrum* | 0 | 0 | 0 | 0 | 0 | 0 | 0 | 0 | 0 | 0 | 1 | 0 | 1 | 0 | 4 | 0 |
| *Certhionyx variegatus* | 5 | 0 | 39 | 10 | 0 | 3 | 1 | 0 | 0 | 0 | 0 | 0 | 3 | 5 | 1 | 0 |
| *Manorina flavigula* | 5 | 8 | 10 | 3 | 0 | 10 | 0 | 10 | 18 | 14 | 3 | 3 | 0 | 8 | 16 | 33 |
| *Epthianura tricolor* | 0 | 0 | 0 | 1 | 0 | 3 | 8 | 1 | 11 | 2 | 3 | 11 | 0 | 119 | 1 | 5 |
| *Melanodryas cucullata* | 0 | 2 | 0 | 0 | 0 | 2 | 2 | 0 | 3 | 2 | 6 | 2 | 3 | 8 | 3 | 12 |
| *Petroica goodenovii* | 3 | 1 | 5 | 5 | 18 | 14 | 9 | 22 | 13 | 17 | 14 | 18 | 5 | 22 | 6 | 15 |
| *Pomatostomus superciliosus* | 7 | 21 | 37 | 7 | 37 | 6 | 71 | 7 | 58 | 11 | 60 | 11 | 53 | 6 | 67 | 13 |
| *Pomatostomus temporalis* | 48 | 11 | 20 | 13 | 8 | 1 | 14 | 3 | 32 | 0 | 0 | 0 | 5 | 15 | 0 | 1 |
| *Daphoenositta chrysoptera* | 0 | 0 | 0 | 0 | 0 | 0 | 0 | 0 | 0 | 0 | 8 | 9 | 0 | 0 | 0 | 0 |
| *Oreoica gutturalis* | 1 | 2 | 0 | 0 | 0 | 0 | 0 | 0 | 0 | 0 | 0 | 2 | 0 | 11 | 6 | 4 |
| *Pachycephala rufiventris* | 29 | 12 | 26 | 4 | 24 | 22 | 35 | 19 | 11 | 17 | 31 | 31 | 30 | 45 | 34 | 20 |
| *Colluricincla harmonica* | 8 | 0 | 10 | 0 | 9 | 4 | 16 | 6 | 14 | 6 | 19 | 2 | 32 | 9 | 35 | 5 |
| *Rhipidura albiscapa* | 0 | 1 | 0 | 0 | 0 | 0 | 0 | 0 | 1 | 0 | 1 | 0 | 0 | 0 | 0 | 1 |
| *Rhipidura leucophrys* | 31 | 15 | 27 | 7 | 38 | 21 | 41 | 20 | 24 | 17 | 42 | 41 | 80 | 54 | 62 | 43 |
| *Grallina cyanoleuca* | 49 | 39 | 12 | 23 | 31 | 20 | 31 | 28 | 11 | 27 | 57 | 29 | 121 | 136 | 58 | 60 |
| *Lalage sueurii* | 4 | 8 | 52 | 41 | 3 | 22 | 11 | 14 | 10 | 17 | 17 | 33 | 60 | 182 | 9 | 32 |
| *Coracina novaehollandiae* | 22 | 22 | 21 | 16 | 12 | 23 | 23 | 12 | 6 | 17 | 13 | 31 | 36 | 64 | 33 | 75 |
| *Coracina maxima* | 0 | 0 | 0 | 0 | 0 | 1 | 0 | 0 | 0 | 0 | 0 | 0 | 0 | 0 | 0 | 0 |
| *Artamus minor* | 0 | 0 | 5 | 0 | 39 | 3 | 0 | 2 | 5 | 0 | 29 | 2 | 0 | 0 | 0 | 0 |
| *Artamus personatus* | 0 | 0 | 1 | 0 | 0 | 0 | 3 | 0 | 0 | 0 | 5 | 0 | 25 | 82 | 12 | 2 |
| *Artamus superciliosus* | 0 | 0 | 0 | 0 | 0 | 0 | 2 | 0 | 0 | 0 | 0 | 0 | 0 | 0 | 0 | 0 |
| *Artamus cinereus* | 4 | 3 | 5 | 4 | 0 | 19 | 25 | 21 | 1 | 35 | 2 | 38 | 1 | 67 | 4 | 16 |
| *Cracticus torquatus* | 0 | 0 | 1 | 1 | 0 | 0 | 0 | 0 | 0 | 2 | 0 | 0 | 0 | 0 | 0 | 0 |
| *Cracticus nigrogularis* | 0 | 15 | 10 | 11 | 5 | 12 | 8 | 11 | 3 | 2 | 3 | 5 | 4 | 9 | 2 | 2 |
| *Cracticus tibicen* | 0 | 12 | 0 | 10 | 1 | 20 | 0 | 3 | 1 | 8 | 1 | 2 | 0 | 5 | 3 | 4 |
| *Corvus bennetti* | 0 | 0 | 0 | 0 | 0 | 4 | 0 | 3 | 1 | 0 | 0 | 0 | 2 | 0 | 17 | 1 |
| *Corvus orru* | 0 | 14 | 5 | 27 | 0 | 0 | 2 | 4 | 5 | 2 | 7 | 5 | 14 | 4 | 0 | 2 |
| *Ptilonorhynchus guttata* | 3 | 9 | 11 | 9 | 10 | 10 | 15 | 14 | 9 | 18 | 11 | 12 | 7 | 11 | 14 | 18 |
| *Emblema pictum* | 0 | 0 | 0 | 0 | 0 | 0 | 0 | 1 | 0 | 22 | 26 | 0 | 0 | 0 | 0 | 0 |
| *Taeniopygia guttata* | 279 | 26 | 201 | 64 | 208 | 79 | 306 | 293 | 425 | 272 | 510 | 253 | 229 | 897 | 182 | 345 |
| *Dicaeum hirundinaceum* | 137 | 59 | 127 | 48 | 89 | 61 | 96 | 49 | 165 | 54 | 96 | 45 | 57 | 29 | 64 | 40 |
| *Cheramoeca leucosterna* | 1 | 0 | 0 | 6 | 0 | 0 | 0 | 5 | 0 | 0 | 0 | 2 | 0 | 1 | 0 | 2 |
| *Petrochelidon ariel* | 0 | 10 | 0 | 0 | 0 | 3 | 0 | 2 | 3 | 0 | 0 | 0 | 23 | 12 | 0 | 0 |
| *Anthus australis* | 0 | 0 | 0 | 3 | 2 | 0 | 1 | 0 | 1 | 2 | 4 | 1 | 1 | 1 | 1 | 3 |
| *Megalurus cruralis* | 0 | 0 | 0 | 0 | 0 | 0 | 0 | 0 | 0 | 0 | 0 | 0 | 0 | 9 | 0 | 1 |
| *Megalurus mathewsi* | 0 | 4 | 10 | 52 | 0 | 11 | 2 | 5 | 0 | 7 | 0 | 5 | 20 | 67 | 8 | 52 |

Table S2: Total species counts for each plot over the entire survey period.

|  |  | I1 | I2 | I3 | I4 | I5 | N1 | N2 | N3 | N4 | N5 |
| --- | --- | --- | --- | --- | --- | --- | --- | --- | --- | --- | --- |
| Stubble Quail | *Coturnix pectoralis* | 0 | 0 | 0 | 0 | 0 | 0 | 0 | 1 | 0 | 0 |
| Brown Quail | *Coturnix ypsilophora* | 0 | 0 | 2 | 0 | 8 | 0 | 0 | 1 | 1 | 0 |
| Wedge-tailed Eagle | *Aquila audax* | 0 | 0 | 0 | 1 | 0 | 2 | 0 | 0 | 0 | 1 |
| Black-shouldered Kite | *Elanus axillaris* | 0 | 0 | 0 | 0 | 0 | 0 | 1 | 21 | 0 | 0 |
| Black Kite | *Milvus migrans* | 2 | 2 | 2 | 3 | 6 | 3 | 16 | 20 | 2 | 0 |
| Whistling Kite | *Haliastur sphenurus* | 1 | 2 | 0 | 1 | 4 | 2 | 1 | 1 | 0 | 0 |
| Brown Goshawk | *Accipter fasciatus* | 0 | 4 | 5 | 5 | 5 | 1 | 12 | 2 | 0 | 0 |
| Collared Sparrowhawk | *Accipter cirrocephalus* | 1 | 1 | 2 | 1 | 1 | 0 | 1 | 1 | 0 | 2 |
| Black Falcon | *Falco subniger* | 0 | 0 | 0 | 0 | 0 | 1 | 1 | 2 | 2 | 0 |
| Brown Falcon | *Falco berigora* | 3 | 1 | 1 | 2 | 3 | 4 | 11 | 16 | 1 | 0 |
| Nankeen Kestrel | *Falco cenchroides* | 1 | 0 | 0 | 1 | 5 | 4 | 0 | 6 | 0 | 0 |
| Peregrine Falcon | *Falco peregrinus* | 1 | 0 | 0 | 0 | 0 | 0 | 0 | 0 | 0 | 0 |
| Australian Hobby | *Falco longipennis* | 0 | 0 | 0 | 1 | 1 | 0 | 0 | 1 | 0 | 0 |
| Little Button Quail | *Turnix velox* | 0 | 0 | 0 | 1 | 7 | 13 | 0 | 19 | 12 | 6 |
| Bush Stone-curlew | *Burhinus grallarius* | 0 | 57 | 0 | 1 | 0 | 0 | 0 | 0 | 0 | 0 |
| Peaceful Dove | *Geopelia placida* | 1 | 26 | 3 | 7 | 1 | 0 | 9 | 2 | 1 | 0 |
| Diamond Dove | *Geopelia cuneata* | 63 | 47 | 56 | 70 | 129 | 72 | 112 | 55 | 258 | 153 |
| Common Bronzewing | *Phaps chalcoptera* | 10 | 2 | 19 | 3 | 5 | 6 | 2 | 6 | 3 | 2 |
| Crested Pigeon | *Ochyphaps lophotes* | 35 | 126 | 260 | 874 | 107 | 31 | 166 | 182 | 194 | 13 |
| Spinifex Pigeon | *Geophaps plumifera* | 3 | 0 | 1 | 0 | 0 | 0 | 0 | 1 | 0 | 6 |
| Tawny Frogmouth | *Podargus strigoides* | 0 | 0 | 0 | 0 | 0 | 0 | 0 | 1 | 0 | 0 |
| Spotted Nightjar | *Eurostopodus argus* | 4 | 0 | 0 | 0 | 0 | 0 | 0 | 0 | 0 | 3 |
| Red-tailed Black-cockatoo | *Calyptorhynchus banksii* | 0 | 0 | 0 | 2 | 0 | 0 | 0 | 11 | 0 | 2 |
| Galah | *Eolophus roseicapilla* | 16 | 105 | 125 | 196 | 91 | 19 | 150 | 304 | 2 | 0 |
| Little Corella | *Cacatua sanguinea* | 0 | 0 | 0 | 0 | 0 | 0 | 2 | 0 | 0 | 0 |
| Cockatiel | *Nymphicus hollandicus* | 0 | 0 | 0 | 5 | 0 | 0 | 1 | 7 | 0 | 0 |
| Budgerigar | *Melopsittacus undulatus* | 74 | 242 | 172 | 94 | 388 | 587 | 328 | 448 | 90 | 41 |
| Australian ringneck | *Barnadius zonarius* | 41 | 166 | 151 | 129 | 100 | 76 | 174 | 360 | 39 | 23 |
| Mulga Parrot | *Psephotus varius* | 8 | 4 | 3 | 2 | 5 | 4 | 2 | 0 | 0 | 0 |
| Pallid Cuckoo | *Cacomantis pallidus* | 0 | 0 | 0 | 0 | 0 | 4 | 2 | 3 | 3 | 3 |
| Black-eared Cuckoo | *Chalcites osculans* | 0 | 0 | 0 | 3 | 0 | 1 | 0 | 0 | 2 | 2 |
| Horsfield's Bronze Cuckoo | *Chalcites basalis* | 3 | 0 | 5 | 5 | 0 | 1 | 0 | 5 | 12 | 2 |
| Channel-billed Cuckoo | *Scythrops novaehollandiae* | 0 | 0 | 0 | 0 | 0 | 0 | 2 | 0 | 0 | 0 |
| Red-backed Kingfisher | *Todiramphus pyrrhopygius* | 1 | 1 | 1 | 2 | 21 | 12 | 70 | 52 | 20 | 2 |
| Sacred Kingfisher | *Todiramphus sanctus* | 0 | 1 | 1 | 1 | 0 | 2 | 3 | 3 | 2 | 0 |
| Rainbow Bee-eater | *Merops ornatus* | 0 | 4 | 9 | 3 | 14 | 45 | 78 | 216 | 3 | 3 |
| Splendid Fairy-wren | *Malurus splendens* | 200 | 12 | 290 | 126 | 225 | 200 | 17 | 92 | 475 | 212 |
| Variegated Fairy-wren | *Malurus lamberti* | 12 | 30 | 61 | 25 | 2 | 9 | 23 | 24 | 29 | 47 |
| Red-browed Pardalote | *Pardalotus rubricatus* | 1 | 0 | 2 | 0 | 0 | 1 | 5 | 10 | 0 | 0 |
| Striated Pardalote | *Pardalotus striatus* | 1 | 1 | 0 | 1 | 0 | 4 | 0 | 2 | 0 | 0 |
| Redthroat | *Pyrrholaemus brunneus* | 7 | 0 | 0 | 0 | 0 | 1 | 0 | 0 | 6 | 5 |
| Western Gerygone | *Gerygone fusca* | 24 | 1 | 7 | 6 | 6 | 32 | 6 | 4 | 21 | 8 |
| Weebill | *Smicrornis brevirostris* | 2 | 2 | 87 | 13 | 51 | 15 | 6 | 20 | 12 | 2 |
| Yellow-rumped Thornbill | *Acanthiza chrysorrhoa* | 65 | 0 | 21 | 52 | 31 | 81 | 9 | 85 | 75 | 78 |
| Inland Thornbill | *Acanthiza apicalis* | 77 | 0 | 4 | 22 | 7 | 41 | 0 | 36 | 133 | 100 |
| Chestnut-rumped Thornbill | *Acanthiza uropygialis* | 13 | 2 | 4 | 5 | 3 | 18 | 0 | 9 | 50 | 4 |
| Slaty-backed Thornbill | *Acanthiza robustirostris* | 6 | 0 | 0 | 1 | 0 | 0 | 0 | 0 | 0 | 7 |
| Southern Whiteface | *Aphelocephala leucopsis* | 1 | 0 | 0 | 0 | 0 | 2 | 0 | 0 | 0 | 0 |
| Spiny-cheeked Honeyeater | *Acanthagenys rufogularis* | 144 | 356 | 630 | 518 | 261 | 94 | 206 | 165 | 152 | 66 |
| Singing Honeyeater | *Lichenostomus virescens* | 72 | 47 | 291 | 289 | 198 | 41 | 147 | 80 | 101 | 74 |
| Grey-headed Honeyeater | *Lichenostomus keartlandi* | 25 | 17 | 25 | 25 | 19 | 0 | 7 | 17 | 11 | 1 |
| White-plumed Honeyeater | *Lichenostomus penicillatus* | 27 | 913 | 562 | 422 | 135 | 22 | 303 | 54 | 13 | 7 |
| Brown Honeyeater | *Lichmera indistincta* | 122 | 76 | 375 | 368 | 58 | 8 | 69 | 18 | 84 | 38 |
| White-fronted Honeyeater | *Pumella albifrons* | 0 | 3 | 8 | 1 | 0 | 0 | 4 | 2 | 1 | 1 |
| Grey Honeyeater | *Conopophila whitei* | 2 | 0 | 0 | 0 | 1 | 0 | 4 | 0 | 0 | 0 |
| Black Honeyeater | *Sugomel nigrum* | 0 | 0 | 2 | 2 | 2 | 0 | 0 | 0 | 0 | 0 |
| Pied Honeyeater | *Certhionyx variegatus* | 9 | 0 | 15 | 20 | 5 | 1 | 1 | 0 | 11 | 5 |
| Yellow-throated Miner | *Manorina flavigula* | 22 | 8 | 8 | 10 | 4 | 3 | 25 | 60 | 1 | 0 |
| Crimson Chat | *Epthianura tricolor* | 0 | 5 | 11 | 5 | 2 | 96 | 0 | 16 | 10 | 20 |
| Hooded Robin | *Melanodryas cucullata* | 2 | 0 | 3 | 7 | 5 | 2 | 2 | 3 | 5 | 16 |
| Red-capped Robin | *Petroica goodenovii* | 31 | 0 | 15 | 10 | 17 | 12 | 0 | 3 | 40 | 59 |
| White-browed Babbler | *Pomatostomus superciliosus* | 45 | 60 | 106 | 112 | 67 | 0 | 10 | 3 | 62 | 7 |
| Grey-crowned Babbler | *Pomatostomus temporalis* | 25 | 54 | 8 | 27 | 13 | 5 | 14 | 17 | 0 | 8 |
| Varied Sittella | *Daphoenositta chrysoptera* | 0 | 0 | 0 | 0 | 8 | 0 | 3 | 6 | 0 | 0 |
| Crested Bellbird | *Oreoica gutturalis* | 0 | 1 | 3 | 2 | 1 | 8 | 0 | 3 | 6 | 2 |
| Rufous Whistler | *Pachycephala rufiventris* | 77 | 12 | 52 | 52 | 27 | 21 | 17 | 12 | 54 | 66 |
| Grey Shrike-thrush | *Colluricincla harmonica* | 12 | 49 | 24 | 44 | 14 | 1 | 13 | 6 | 5 | 7 |
| Grey Fantail | *Rhipidura albiscapa* | 1 | 0 | 1 | 0 | 0 | 0 | 0 | 0 | 0 | 2 |
| Willie Wagtail | *Rhipidura leucophrys* | 45 | 68 | 88 | 92 | 52 | 31 | 77 | 54 | 24 | 32 |
| Magpie Lark | *Grallina cyanoleuca* | 6 | 132 | 73 | 73 | 86 | 62 | 126 | 123 | 20 | 31 |
| White-winged Triller | *Lalage sueurii* | 3 | 3 | 47 | 82 | 31 | 98 | 37 | 157 | 11 | 46 |
| Black-faced Cuckoo-shrike | *Coracina novaehollandiae* | 9 | 49 | 36 | 49 | 23 | 23 | 61 | 140 | 24 | 12 |
| Ground Cuckoo-shrike | *Coracina maxima* | 0 | 0 | 0 | 0 | 0 | 0 | 1 | 0 | 0 | 0 |
| Little Woodswallow | *Artamus minor* | 19 | 0 | 7 | 28 | 24 | 2 | 0 | 2 | 0 | 3 |
| Masked Woodswallow | *Artamus personatus* | 0 | 1 | 8 | 15 | 22 | 26 | 3 | 25 | 0 | 30 |
| White-browed Woodswallow | *Artamus superciliosus* | 0 | 0 | 0 | 2 | 0 | 0 | 0 | 0 | 0 | 0 |
| Black-faced Woodswallow | *Artamus cinereus* | 0 | 0 | 5 | 8 | 29 | 53 | 28 | 116 | 4 | 2 |
| Grey Butcherbird | *Cracticus torquatus* | 1 | 0 | 0 | 0 | 0 | 0 | 0 | 1 | 2 | 0 |
| Pied Butcherbird | *Cracticus nigrogularis* | 4 | 3 | 6 | 16 | 6 | 32 | 3 | 25 | 3 | 4 |
| Australian Magpie | *Cracticus tibicen* | 1 | 1 | 0 | 0 | 4 | 10 | 6 | 42 | 4 | 2 |
| Little Crow | *Corvus bennetti* | 0 | 9 | 1 | 2 | 8 | 4 | 3 | 0 | 1 | 0 |
| Torresian Crow | *Corvus orru* | 1 | 19 | 3 | 4 | 6 | 6 | 4 | 48 | 0 | 0 |
| Western Bowerbird | *Ptilonorhynchus guttata* | 5 | 12 | 10 | 29 | 24 | 12 | 5 | 11 | 47 | 26 |
| Painted Finch | *Emblema pictum* | 13 | 13 | 0 | 0 | 0 | 0 | 23 | 0 | 0 | 0 |
| Zebra Finch | *Taeniopygia guttata* | 395 | 444 | 243 | 611 | 647 | 412 | 705 | 464 | 355 | 293 |
| Mistletoebird | *Dicaeum hirundinaceum* | 82 | 88 | 234 | 281 | 146 | 21 | 114 | 98 | 113 | 39 |
| White-backed Swallow | *Cheramoeca leucosterma* | 1 | 0 | 0 | 0 | 0 | 0 | 8 | 8 | 0 | 0 |
| Fairy Martin | *Petrochelidon ariel* | 0 | 0 | 0 | 0 | 26 | 8 | 0 | 19 | 0 | 0 |
| Australian Pipit | *Anthus australis* | 0 | 0 | 0 | 1 | 9 | 6 | 0 | 4 | 0 | 0 |
| Brown Songlark | *Megalurus cruralis* | 0 | 0 | 0 | 0 | 0 | 0 | 1 | 9 | 0 | 0 |
| Rufous Songlark | *Megalurus mathewsi* | 1 | 4 | 2 | 7 | 26 | 25 | 64 | 91 | 22 | 1 |
